# Supplementary material for: Amaranthus cruentus L. Seed Oil Counteracts UVA-Radiation-Induced Inhibition of Collagen Biosynthesis and Wound Healing in Human Skin Fibroblasts
Source: Int J Mol Sci. 2024 Jan 11;25(2):925. doi: 10.3390/ijms25020925 (PMC10815470; doi:10.3390/ijms25020925)
Supplement: Supplementary file 1 [file ijms-25-00925-s001.zip › ijms-2787783-File S1.pdf]

File S1. Material Safety Data Sheet for tested oil from *Amaranthus cruentus* seed [source: Szarłat M. i W. Lenkiewicz Zawady Poland].

**Product Specification**	**Product Name:**<br>**Cosmetic Amaranth Oil**	**Specification Number:**<br>**SP-10/OL**
INCI Name:	*Amaranthus cruentus* seed oil	
Product Description	Unrefined oil obtained by cold-pressing the seeds of *Amaranthus cruentus*. Crystallization below 17°C is a natural phenomenon and does not affect the quality of the product.	
Country of Origin	Poland	
Intended Use	For external use, for massage, as an additive to baths	
Ingredients	100% amaranthus seed oil	
Physical and Chemical Properties:	Color	Yellow to orange
	Odor	Specific, without foreign odors
	Density (g/cm^3)	0.92
	Water and Volatile Substances Content (% max)	0.5
	Acid Value (mg KOH/g of oil) max	5.0
	Peroxide Value (mEq O/kg of oil) max	10.0
	Insoluble Impurities Content (% max)	0.2
	* Iodine value (g I_2/100)	120-140
Harmful Metals	* Lead (mg/kg) max:	0.1
Fatty Acid Content (%)*	Palmitic C16:0	15.0-21.2
	Stearic C18:0	2.5-5.5
	Oleic C18:1	15.5-22.5
	Linoleic C18:2	42.0-65.0
Other:	Squalene Content (%)*	4.0-8.0
Microbiological Requirements*	Presence of *Escherichia coli* in 1g<br>Presence of *Staphylococcus aureus* in 1g<br>Presence of *Pseudomonas aeruginosa* in 1g<br>Presence of *Candida albicans* in 1g<br>Total Count of Mesophilic Aerobic Microorganisms CFU/g max:	Absent<br>Absent<br>Absent<br>Absent<br>$5x10^3$
Allergens	Does not contain**	
GMO Information	Does not contain genetically modified ingredients	
Packaging	Allowed for contact with cosmetics, clean, undamaged, ensuring proper quality and durability of the product	
Labeling	Labeling of individual packaging in accordance with the current requirements contained in the Cosmetics Act of March 30, 2001 (Journal of Laws No. 42, item 473)	
Storage Conditions	Rooms should be dry, clean, free from foreign odors and pests; optimal temperature not exceeding 20°C; optimal humidity not exceeding 80%	
Shelf Life	12 months from the date of production	
Transportation Conditions	Clean transport vehicles free from foreign odors; products transported in sealed, properly secured packaging	

This specification is developed based on internal standards and current cosmetics regulations.
*Periodic testing conducted once a year
** According to Annex III of Regulation 1223/2009/EC dated November 30, 2009, on cosmetic products.
