# Supplementary material for: Amaranthus cruentus L. Seed Oil Counteracts UVA-Radiation-Induced Inhibition of Collagen Biosynthesis and Wound Healing in Human Skin Fibroblasts
Source: Int J Mol Sci. 2024 Jan 11;25(2):925. doi: 10.3390/ijms25020925 (PMC10815470; doi:10.3390/ijms25020925)
Supplement: Supplementary file 1 [file ijms-25-00925-s001.zip › ijms-2787783-Supplementary Materials 2.pdf]

Table S1 Collagen biosynthesis measurements in fibroblasts irradiated with UVA and treated with AmO at concentrations of 0.05%. 0.1% and 0.15%. (1st - first measurement in sample. 2nd - second measurement in sample. Δ - difference between 1st and 2nd measurement in sample. *statistically significant differences at $p < 0.05$ compared to the control. ##statistically significant difference at $p < 0.05$ compared to UVA. SD – standard deviation. SEM – standard error).

Sample	1st	2nd	Δ^1	1st	2nd	Δ^2	1st	2nd	Δ^3	Mean value (Δ^1+ Δ^2+ Δ^3)/3	% per cent	SD	SEM
**Control**	2486	1503	**983**	1849	1682	**167**	1866	1863	**3**	384.33	100	524.90	399.11
**0.05% AmO**	1611	1387	**224**	1542	1321	**221**	2031	1379	**652**	365.66	95.14	247.97	190.88
**0.1% AmO**	2296	1752	**544**	2115	1753	**362**	2069	1892	**177**	361	93.93*	183.50	122.66
**0.15%AmO**	2286	1722	**564**	2015	1743	**272**	2079	1882	**197**	344.33	89.59	193.89	146.44
**UVA**	914	616	**298**	846	681	**165**	685	517	**198**	220.33	57.32*	69.25	51.777
**0.05% AmO + UVA**	1182	948	**234**	1156	1035	**121**	1400	862	**538**	297.66	77.45##	215.66	160.22
**0.1% AmO + UVA**	2286	1822	**464**	2115	1843	**272**	1979	1782	**197**	311	80.91##	137.70	102
**0.15%AmO + UVA**	2186	1922	**264**	2005	1543	**462**	2129	1882	**247**	324.33	84.38##	119.52	91.777

Table S2 Prolidase activity in fibroblasts irradiated with UVA and treated with AmO at concentrations of 0.05%. 0.1% and 0.15%. The mean values from the experiments performed in triplicates. *statistically significant differences at $p < 0.05$ compared with the control. ##statistically significant difference at $p < 0.05$ compared to UVA.

Sample	Control	0.05% AmO	0.1% AmO	0.15%AmO	UVA	0.05% AmO + UVA	0.1% AmO + UVA	0.15%AmO + UVA
**Mean value**	116.4	123.38	126.87	130.36	62.86	87.3	93.12	98.94
**% of control value**	100	106	109	112	54*	75##	80##	85##

Figure S1 Western blot densitometry (plot measurement) results by gel analysis tool for ImageJ® 1.8.0 for β1- integrin (A) and IGF-IR (B) receptors expression in UVA-irradiated fibroblasts in the presence of AmO at concentrations of 0.05%. 0.1%. and 0.15%.

(A)

Sample	Plot measurement	% of control
**Control (a)**	7581.518	100
**0.05% AmO (b)**	6555.539	86.46737
**0.1% AmO (c)**	6438.468	84.9232
**0.15%AmO (d)**	6348.63	83.73824
**UVA (e)**	1439.355	18.98505
**0.05% AmO + UVA (f)**	1788.941	23.59608
**0.1% AmO + UVA (g)**	3641.104	48.02605
**0.15%AmO + UVA (h)**	3841.083	50.66377

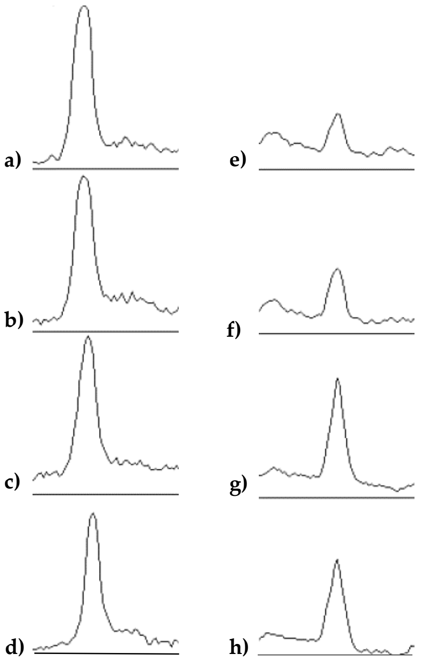

(B)

Sample	Plot measurement	% of control
**Control (a)**	12890.782	100
**0.05% AmO (b)**	11842.539	91.86827
**0.1% AmO (c)**	11883.075	92.18273
**0.15%AmO (d)**	12334.953	95.68817
**UVA (e)**	3148.154	24.42175
**0.05% AmO + UVA (f)**	7389.397	57.32311
**0.1% AmO + UVA (g)**	8502.589	65.95867
**0.15%AmO + UVA (h)**	8557.125	66.38174

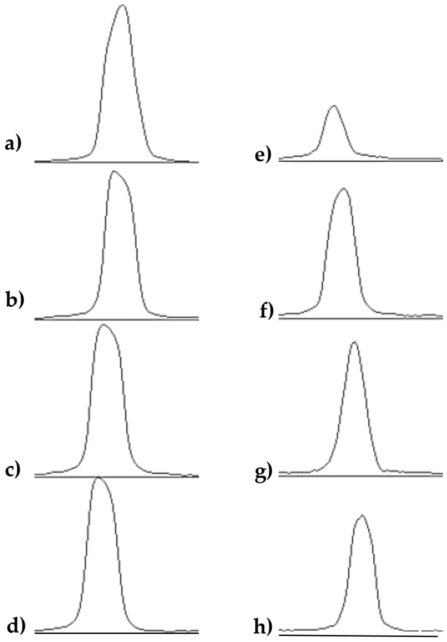

Figure S2 Western blot densitometry (plot measurement) results by gel analysis tool for ImageJ® 1.8.0 for TGF-β1 (A) and p38 (B) protein expression in UVA-irradiated fibroblasts in the presence of AmO at concentrations of 0.05%. 0.1%. and 0.15%.

(A)

Sample	Plot measurement	% of control
**Control [(a)]**	4187.0	100
**0.05% AmO [(b)]**	5067.7	121
**0.1% AmO [(c)]**	5880.4	170
**0.15%AmO [(d)]**	6208.1	252
**UVA [(e)]**	640.5	15
**0.05% AmO + UVA [(f)]**	1068.4	26
**0.1% AmO + UVA [(g)]**	2060.9	49
**0.15%AmO + UVA [(h)]**	3273.3	78

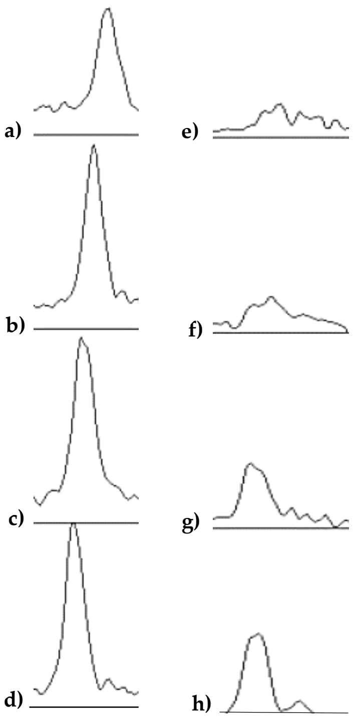

B)

Sample	Plot measurement	% of control
**Control [(a)]**	1473.941	100
**0.05% AmO [(b)]**	1520.719	103
**0.1% AmO [(c)]**	1497.548	102
**0.15%AmO [(d)]**	1823.406	124
**UVA [(e)]**	4745.033	322
**0.05% AmO + UVA [(f)]**	3081.426	209
**0.1% AmO + UVA [(g)]**	2911.205	198
**0.15%AmO + UVA [(h)]**	1548.184	105

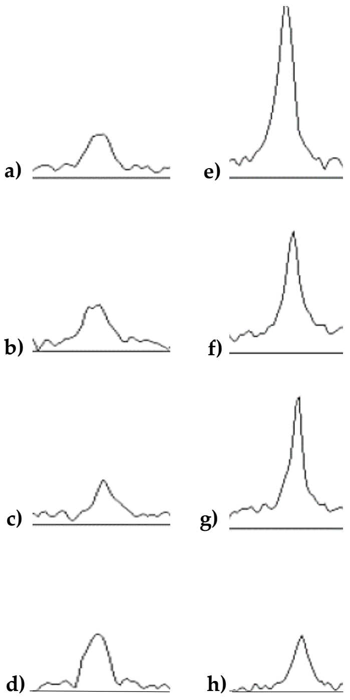

Figure S3 Western blot densitometry (plot measurement) results by gel analysis tool for ImageJ® 1.8.0 for COX-2 protein expression in UVA-irradiated fibroblasts in the presence of AmO at concentrations of 0.05%. 0.1%. and 0.15%.

Sample	Plot measurement	% of control
**Control** (a)	2926.255	100
**0.05% AmO** (b)	3177.255	109
**0.1% AmO** (c)	3385.983	116
**0.15%AmO** (d)	3484.154	119
**UVA** (e)	7350.811	251
**0.05% AmO + UVA** (f)	6140.983	210
**0.1% AmO + UVA** (g)	5932.225	203
**0.15%AmO + UVA** (h)	2874.418	98

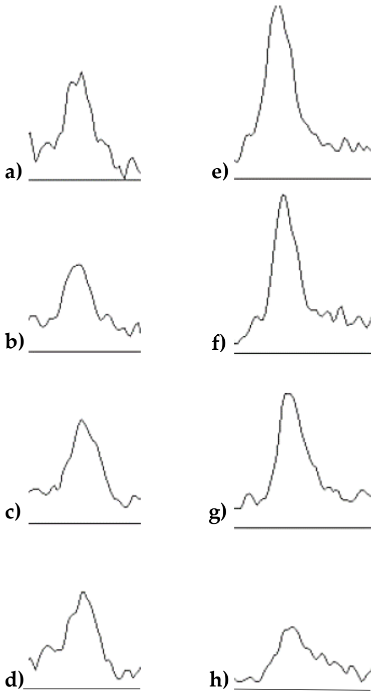

c)
